# Costs and cost-effectiveness of the Kerala pilot screening programme for diabetic retinopathy in the public health system

**DOI:** 10.1038/s41433-024-03304-w

**Published:** 2024-09-03

**Authors:** Raphael Wittenberg, Robert Anderson, Stuart Redding, Bipin Gopal, Rajeev Sadanandan, Vasudeva Iyer Sahasranamam, Simon George, Lakshmi Premnazir, Gopalakrishnan Netuveli, Jyotsna Srinath, Radha Ramakrishnan, Dolores Conroy, Sobha Sivaprasad

**Affiliations:** 1 Nuffield Department of Primary Care Health Sciences, University of Oxford, Oxford, UK; 2 Directorate of Health Services, Thiruvananthapuram, Kerala India; 3 Health Systems Transformation Platform, New Delhi, India; 4 Regional Institute of Ophthalmology, Thiruvananthapuram, Kerala India; 5 Institute for Connected Communities, University of East London, London, UK; 6 Vision Sciences, UCL Institute of Ophthalmology, London, UK; 7 NIHR Biomedical Research Centre, UCL and Moorfields Eye Hospital NHS Foundation Trust, London, UK

**Keywords:** Health care economics, Eye diseases

## Abstract

**Background/objectives:**

The Government of Kerala initiated a pilot screening programme for diabetic retinopathy in 16 Family Health Centres in Thiruvananthapuram district in 2019 in collaboration with the ORNATE India project. The evaluation of this pilot included a study of its costs and cost-effectiveness to inform decisions about extending the programme throughout Kerala.

**Subjects/methods:**

The participants comprise all 5307 people who were screened for diabetic retinopathy under the pilot programme for whom data could be collected.

**Results:**

The costs of the pilot programme are estimated at INR 11.3 million (including INR 1.9 million costs to individuals) and the benefits at 514 QALYs, slightly over one QALY per person treated. The cost per QALY was INR 22,000, which is well below India’s Gross National Income per person.

**Conclusions:**

Kerala’s 2019 pilot screening programme for diabetic retinopathy was highly cost-effective.

## Introduction

Diabetic retinopathy (DR), a common complication of diabetes, is a leading preventable cause of visual impairment and blindness. The condition is mostly asymptomatic and late presentation can result is vision impairment, with its associated loss of quality of life and economic productivity. Screening for sight threatening DR (STDR) and prompt treatment can reduce the extent of visual impairment. As the numbers of older people continue to rise, the numbers with diabetes and its complications including DR are expected to continue to increase worldwide; and the burden of diabetes and its complications are increasingly moving towards Lower- and Middle-Income Countries (LMICs) [1]. It is therefore becoming ever more important to promote measures to detect and treat DR by establishing DR screening programmes and treatment pathways before people with this condition lose their sight. This is however more challenging in LMICs that have less well funded and developed health care systems.

There were an estimated 77 million adults (aged 20–79 years) with diabetes in India in 2019, and this number is projected to increase to 100.95 million in 2030 and 134.23 million in 2045 [2]. An additional 43.9 million people are estimated to have undiagnosed diabetes [2]. The age-adjusted prevalence of diabetes in India is projected to rise from 10.4 to 11.2% in 2030 and to 11.5% in 2045. Due to this increasing prevalence of diabetes in India, DR is rising among working adults in India. It is expected to increase from 4.21 million in 2020 to 6.08 million by 2030 [3]. An estimated 472 billion INR and 2.86 million quality-adjusted life years (QALYs) are lost annually in India due to blindness and moderate to severe visual impairment among people aged 40 and over with diabetes [4].

As in most LMICs, there are no systematic national or state-wide screening programmes for DR in India. Retinal examinations or photography are performed opportunistically when people with diabetes visit an eye facility, though often only after vision loss [5].

Kerala is the most advanced state in India in terms of health, literacy and economy but it also has a high prevalence of diabetes. The Government of Kerala launched the Aardram Mission in 2017 to transform the State’s public health care system to achieve the Sustainable Development Goals (SDGs). The overarching objectives included providing equitable, affordable and quality care to citizens from all socio-economic strata, strengthening the public care system by decentralising healthcare to primary care-led services and initiating preventive medicine to address the impact of non-communicable diseases, especially hypertension and diabetes [6].

The prevalence of diabetes in Kerala is 24.8% for men and 27.0% for women, according to the National Family Health Survey 5 [7], with an estimated 4% having STDR. Given the urgent need for identifying and treating STDR to decrease the risk of blindness in people with diabetes, the Government of Kerala instituted in 2019 a pilot screening programme for DR, known as the Nayanamritham project [6]. The aim was to understand how the pilot DR care pathway could be scaled up and sustained in the whole of Kerala. The pilot offered screening to people attending diabetes clinics in all the 16 Family Health Centres (FHCs) in Thiruvanathapuram District. Retinal images were taken and transferred to specialist grading staff. Patients whose images indicated eye problems or could not be graded were referred for eye examination [6]. These would lead to treatment for DR where required or treatment for other eye conditions especially cataract surgery. The benefits of the pilot programme therefore extended to treatment for cataract as well as DR.

The analysis reported in this paper, which focuses on the programme’s costs and cost-effectiveness, forms part of the overall evaluation of the pilot. The evaluation is part of the wider ORNATE-India research project [8] which aims to evaluate cost-effective measures for screening for diabetes and its complications and to examine the potential impact of a reduction in the prevalence of blindness on the Indian economy. The project also included an evaluation of a community screening programme for diabetes and its complications in 20 areas of India covering all the six regions, the SMART India Project [6].

## Materials/subjects and methods

### Data collection in Kerala

Data collected by nurses or data operators from the electronic health records (EHR) include age, gender, duration of diabetes, use of insulin, parental history of diabetes, other complications of diabetes (including diabetic kidney disease, cardiovascular complications, and diabetic foot), random blood sugar results, urine dipstick test for albuminuria and blood pressure record. Other study-specific data collected by nurses or data operators on the day of screening include educational status, occupation and income categories, and previous history of DR, cataract surgery or any other ocular history. In addition, they measured body mass index and waist circumference and completed a lifestyle questionnaire on smoking, diet, physical activity, EQ-5D vision bolt-on [9]. The EQ-5D vision bolt-on was used to calculate the quality adjusted life-years and utility value for economic analysis. EQ-5D-5L with vision bolt-on version asks patients to rate their health across 6 dimensions: mobility, self-care, usual activities, pain/discomfort, anxiety/depression and vision. Each dimension is scored in 5 levels: no problems, slight problems, moderate problems, severe problems, and extreme problems. A recent study provided utility values based on the EQ-5D with vision bolt-on. The mapping was done in a clinical trial cohort with macular oedema in central retinal vein occlusion [10].

In the reading centre where the retinal images were examined the data collection included the gradeability of the images, grade of retinopathy in both eyes and presence of cataract. Data collected on patients referred for eye examination included numbers with ungradable images due to cataract, treatment options offered for DR and review appointment.

All those whose data were collected for this study gave their informed consent.

### Other costs and costs coverage

Expert advice based on prices on websites of private clinics in India suggest that the average cost of laser treatment for DR is INR 12,000 over three attendances. Data supplied by the hospitals participating in the study show that the average cost of cataract surgery is INR 18,000. To these figures an estimated figure of INR 950 per attendance at secondary care is added for the costs of travel for the patient and a family member or ASHA accompanying the patient and estimated opportunity cost of the patient’s and family member’s time. The expert advice was provided by senior clinicians from the SMART India study.

The total opportunity costs of the screening programme in the FHCs were calculated as:

- Opportunity cost of the cameras purchased for the screening programme
- Opportunity costs of staff training
- Cost of staff time conducting the screening and grading the images
- Cost of an ASHA worker accompanying the patient

### Markov model description and cost-effectiveness method

To estimate the benefit of treatment for STDR we modelled the progression of STDR using a Markov model with three states – STDR, blindness and death (absorbing state). The annual transition rate from STDR to blindness is 9% without treatment and 2% with treatment [11]. There is assumed to be no recovery from blindness. There is excess mortality among people with diabetes – 1.9 uplift on the age-specific general population mortality rate [11]. There is additional excess mortality in blindness – 2.34 uplift on the general population mortality rate [11]. Data on mortality rates in India for the general older population, people with diabetes and people who are blind are derived from data from the Office of the Registrar General & Census Commissioner [12].

The health-related quality of life (HRQoL) at age 40 with sight threatening DR is 0.70 and with blindness 0.55 [11] on a EuroQoL scale where 1.0 is full health and 0.0 is death. Treatment for STDR therefore produces a HRQoL gain of 0.15 per year. Cataract surgery in India produces a HRQoL gain of 0.2 per year [13]. Lifetime quality-adjusted life years (QALY) gain from cataract surgery was estimated by multiplying this figure by life expectancy modified by a factor assuming an annual decline of HRQoL with age of 0.07% [14].

We conducted a one-way sensitivity analysis of the key inputs to the estimation of the cost-effectiveness of the Kerala pilot screening programme. We assumed a range of 10% above to 10% below the central values for the following variables:

- HRQoL: with STDR, with untreated cataract, with treated cataract, blind,
- Annual percentage rate of decline of HRQoL with treated and untreated cataract,
- Costs: screening cost per person, unit cost of laser photocoagulation, unit cost of cataract surgery,
- Annual transition rates: from STDR to blindness with and without photocoagulation,
- Mortality: excess relative risk of mortality in blind people with diabetes.

We also carried out probability sensitivity analysis (PSA) to test the sensitivity of the incremental cost-effectiveness ratio (ICER) to combined uncertainty in all the inputs. We carried out 10,000 Monte Carlo iterations each of which generates an ICER. Distributions were assigned to each input as follows:

- gamma distribution for inputs with a zero lower bound but no upper bound such as unit costs,
- gamma distribution for the relative risk of mortality among blind people with diabetes,
- beta distribution for inputs whose values lie between zero and unity, such as HRQoL and transition rates.

In each case the variability was represented by a standard deviation (SD) equal to 25% of the point estimate. The large SD value was chosen for two reasons: as a demanding test since the central value of the ICER is a fraction of the willingness-to-pay (wtp) threshold, as explained below, and because the data sources do not provide evidence for the distributions.

The protocol for this study was approved by the Indian Council of Medical Research (2018-0551) dated 13 March 2019.

## Results

### Estimated costs of the screening programme

A total of 5307 patients with diabetes were screened during the course of the 2019 diabetic retinopathy (DR) pilot screening programme at the 16 Family Health Centres (FHCs) included in the pilot project in the Thiruvananthapuram district.

Sixteen cameras were purchased at a cost of INR 300,000 each for the pilot screening programme. The total cost was annuitized on the basis that the cameras will have a lifetime of five years. This produced an estimated annual opportunity cost of slightly over INR one million for 16 cameras, or almost INR 200 per patient screened.

Thirty-two nurses, 16 doctors, 2 graders and 48 ASHA workers received training for conducting the pilot screening programme. The total cost was INR 560,000 including estimated course costs and salary costs, or INR 105 per patient screened.

Each patient screened required on average 12 min of nurse time and one minute of doctor time. These costs were estimated on the basis of staff salaries, with an uplift of 10% to allow for employer oncosts. An average of five minutes of grader’s time was required to grade each patient’s retinal images. The total staff cost was around INR 70 per patient. To this was added an estimated INR 15 for administration and INR 15 for ASHA worker support.

The total cost per patient screened was INR 400 including the annuitized cost of the cameras and of staff training. The total cost of the screening in the 16 FHCs in 2019 was INR 2.14 million. If the pilot programme had continued, the cost might have been lower in the second year, depending on the number of patients screened. While staff turnover would require further staff to be trained in future years, and staff might need refresher training, the cost in subsequent years would likely be lower.

1662 patients – 31% of those screened – were referred to secondary care that included 4 district hospitals (DH) or in a few cases to the Regional Institute of Ophthalmology (RIO). Of these almost half had ungradable retinal images. Comprehensive data are not available on how many of those referred attended a DH or the RIO for an eye examination. Even if such data were available, attendances could have been affected by the pandemic which started less than three months after the 2019 screening pilot ended. We assume as a cautious estimate that 830 patients – 50% of those referred – attended for an eye examination.

Each patient receiving an eye examination at a hospital required on average 10 min of nurse time and 10 min of doctor time. The cost of the staff inputs, plus administration, was INR 205 per patient. To this was added INR 350 travel costs for the patient, INR 350 travel costs for an attendant family member or ASHA worker, INR 100 opportunity cost for the patient and INR 150 opportunity cost for a family member or an ASHA worker. This produced a cost for eye examinations of INR 1155 per patient and total cost of INR 960,000 for 830 patients receiving an eye examination.

The overall estimated economic cost of the Kerala screening pathway was INR 3.1 million, covering the cost of the pathway through to diagnosis but not including the cost of treatment and follow up. This includes costs to patients as well as costs to health services.

### Estimated costs of treatment of cohort screened

Comprehensive data are not available on how many of those who had an eye examination at a DH or the RIO were treated for DR or for cataract. Even if such data were available, the numbers treated could have been affected by the pandemic. We assume on the basis of expert advice that 345 people were treated for DR or would have been treated were it not for the pandemic: this is 70% of those diagnosed with STDR.

We also assume that 160 people were treated, or would have been treated, for cataracts, which is 20% of those with ungradable images. Although the screening programme focused on DR, it identified a proportion of people with cataract who were then operated, treatment which they would usually not otherwise have received. We therefore include cataract surgery as well as DR management in the analysis of the costs and benefits of the pilot.

The cost of laser treatment for 345 patients with STDR is INR 5.1 million and the cost of treating 160 patients for cataract is INR 3.0 million. The estimated total cost of the programme, for the cohort screened during the 2019 pilot, was INR 11.3 million. This includes the costs of the screening at the FHCs, eye examinations at the DH or RIO, and treatment for STDR and cataract but does not include the cost of follow up consultations after treatment or the cost of screening in subsequent years. It amounts to an average cost of the programme per person treated of INR 22,300 for the 505 people assumed to have received laser treatment for STDR or cataract surgery.

Some of the estimated 345 people who received laser treatment may have received treatment in both eyes, but information is not available on the proportion who received bilateral treatment. The health care cost of bilateral treatment would be twice that of treatment of one eye, that is INR 24,000. If, for example 20% received bilateral treatment, the total cost of laser treatment would be almost INR 6.0 million, the total cost of the programme would be INR 12.1 million and the average cost per person treated for STDR or cataract would be INR 29,335.

### Estimated outcome (QALY gain) and cost-effectiveness

We assess the benefits of the pilot in terms of the QALYs gained by these 505 people treated for STDR or cataract. Since there was no routine screening programme in Kerala prior to the pilot, we assume that potentially all these patients would have lost their sight if they had not been screened and treated, albeit a few of them may have received treatment even in the absence of a screening programme. The benefits for those treated therefore comprise both the increase in life expectancy from prevention of sight loss (for treatment of STDR) and the increase in quality of life for the rest of the person’s life from prevention of deterioration to blindness (for treatment of STDR and of cataract). There are also likely to be benefits to the person’s family, but we do not include them due to lack of suitable data.

We model the progression of DR through sight-threatening DR to blindness using a Markov process model, allowing for excess mortality in people with diabetes and additional excess mortality in blindness, as explained above. The benefit from treatment for STDR lies in reducing the annual rate of transition to blindness from 9 to 2% [11], with benefits in terms of quality of life and increased life expectancy. Using these data, mortality data and data from the pilot on the average age of those with STDR – 59 years – we estimate that treatment extends the average remaining life expectancy of patients with STDR from 12.34 years to 12.81 years. We assume that the quality-of-life gain is 0.15 per year, as explained above. We use a discount rate of 3% per year to discount future benefits. On this basis, the estimated discounted lifetime QALY gain from laser treatment for STDR is 0.65 per person treated.

Although cataract is associated with increased mortality [15], we assume that cataract surgery does not increase life expectancy and that its benefit comes entirely from improved quality of life. We assume that the quality-of-life gain from cataract treatment is 0.2 per year, declining slightly over time[13]. We again use a discount rate of 3% per year to discount future benefits. On this basis, the estimated discounted lifetime QALY gain from cataract surgery is 1.81 per person treated.

The pilot screening programme is assumed to have generated 224 QALYs from treatment of STDR (0.65 QALYs each for 324 patients) and 290 QALYs from cataract surgery (1.81 QALYs each for 160 patients). The total QALY gain is 514, an average gain of 1.017 QALYs for the 505 people assumed to have been treated. The incremental cost-effective ratio (ICER) is therefore slightly less than INR 22,000 per QALY. The World Health Organisation (WHO) recommends that an intervention that costs less than the country’s annual GDP per capita per disability-adjusted-life-year (DALY) gained is considered highly cost-effective [16]. Since GDP per head in India is INR 144,000, the cost per QALY of the screening pilot, at around one sixth of GDP per head, puts it firmly into the highly cost-effective range.

The one-way analysis of the sensitivity of this finding to the key assumptions individually found that the main influences on the ICER are the quality-of-life values for health states with and without treatment for STDR or for cataract (Supplemental Fig. 1). The unit costs of treatment have relatively less influence on the ICER. The PSA, which considered the main assumptions jointly, found that the probability of the screening programme being cost-effective is 82% based on the most demanding willingness to pay threshold of INR 144,000 per QALY (Supplemental Figs. 2 and 3). These results, based on our assumptions, demonstrate the robustness of the finding that the Kerala pilot programme of DR screening of people with diabetes followed by treating those diagnosed with STDR or cataract is highly cost effective.

Further details of the sensitivity analyses are presented in the supplementary material.

## Discussion

These findings show that the pilot DR screening programme run in Kerela in 2019 was cost-effective. The programme screened for DR over 5300 people with diabetes in 16 FHCs at an estimated cost of INR 3.1 million for the screening programme and subsequent eye examinations where required or INR 11.3 million including treatment costs. An estimated 505 people received treatment for STDR or cataract surgery following screening and eye examination. Most of them would likely experience severe visual impairment or blindness in the absence of the screening programme. They each gained an estimated average of one QALY. The estimated cost per QALY of INR 22,000 is well within the range for highly cost-effective interventions under WHO guidance on cost-effectiveness thresholds. Sensitivity analyses demonstrate the robustness of the finding that the Kerala pilot programme of DR screening of people with diabetes was highly cost effective. Even if the cost per QALY had been six times higher the Kerala screening pilot would still have been cost-effective.

These findings suggest that a general policy to conduct screening for DR in primary care in India followed by treatment where required would be cost-effective. It would yield a net benefit to Indian society as well as a benefit to people with diabetes at risk of blindness and their families. This finding, which is line with findings from studies of similar DR screening programmes in other LMICs, is likely to be relevant for other LMICs with similar health care infrastructure as India.

There are various technologies that can be used to perform DR screening, and these have varying degrees of cost-effectiveness depending on the environment in which they are used [17, 18]. It is not easy to replicate programmes used in more developed economies due to the lack of infrastructure and geographic features of LMICs [19], but there are examples where screening programmes have been introduced in LMICs.

The findings of this study are broadly similar to those of studies of similar screening programmes in other LMICs, but comparisons need to be treated with some caution: the specific design of programmes may differ, health care systems differ, and socioeconomic circumstances differ between LMICs.

Khan et al. [20] found that a programme for the screening and diagnosis of DR in a primary care setting in South Africa was cost-effective. The cost of the programme, which had a similar pathway to the Kerala pilot, was $1206 per case of blindness averted. Rachapelle et al. [11] found, in their study of a telemedicine DR screening programme in rural Southern India that conducts a one-off screening camp, that the programme was cost-effective ($1320 per QALY) compared with no screening. Vetrini et al. [21] found that ‘annual photographic screening of diabetic patients attending medical diabetes clinics in Malawi, with the provision of laser treatment for those with STDR, appears to be cost-effective in terms of QALYs gained, in our base case scenario’. The cost of the intervention and the years of severe visual impairment averted per patient screened were $209 and 2.2 years respectively.

A strength of this study is that detailed data were collected on those screened in the 16 FHCs covered by the programme despite the challenges of collecting individual data during very busy diabetes clinics. A limitation is that, although the intention was to collect follow up data on eye examinations and treatments for each patient referred for eye examination, this did not in practice prove feasible (other than for a proportion of those referred). Even if it had been feasible, eye examination and treatment for some of those referred were likely delayed due to the Covid19 pandemic.

## Summary

### What was known before

- Diabetic retinopathy (DR), a common complication of diabetes, is a leading preventable cause of visual impairment and blindness.
- As the numbers of older people continue to rise, the numbers with diabetes and its complications including DR are expected to continue to increase worldwide.
- As in most LMICs, there are no systematic national or state-wide screening programmes for DR in India.
- Retinal examinations or photography are performed opportunistically when people with diabetes visit an eye facility, though often only after vision loss.

### What this study adds

- The Government of Kerala instituted in 2019 a pilot screening programme for DR.
- The aim was to understand how the pilot DR care pathway could be scaled up and sustained in the whole of Kerala.
- The analysis reported in this paper focuses on the programme’s costs and cost-effectiveness and forms part of the overall evaluation of the pilot.
- This pilot screening programme for diabetic retinopathy was highly cost-effective and provides a useful model that could be adopted more widely in India and beyond.

## Supplementary information

Supplementary Information. Costs and cost-effectiveness of the Kerala Pilot Screening Programme for Diabetic Retinopathy in the public health system
