# Supplementary material for: Costs and cost-effectiveness of the Kerala pilot screening programme for diabetic retinopathy in the public health system
Source: Eye (Lond). 2024 Sep 3;38(17):3352–6. doi: 10.1038/s41433-024-03304-w (PMC11584660; doi:10.1038/s41433-024-03304-w)
Supplement: Supplementary file 1 — Supplementary Information. Costs and cost-effectiveness of the Kerala Pilot Screening Programme for Diabetic Retinopathy in the public health system [file 41433_2024_3304_MOESM1_ESM.docx]

**Costs and cost-effectiveness of the Kerala Pilot Screening Programme for Diabetic Retinopathy in the public health system**

**Supplementary Information**

**Figure 1 Incremental Cost-effectiveness Ratio (ICER): sensitivity analysis Tornado diagram**


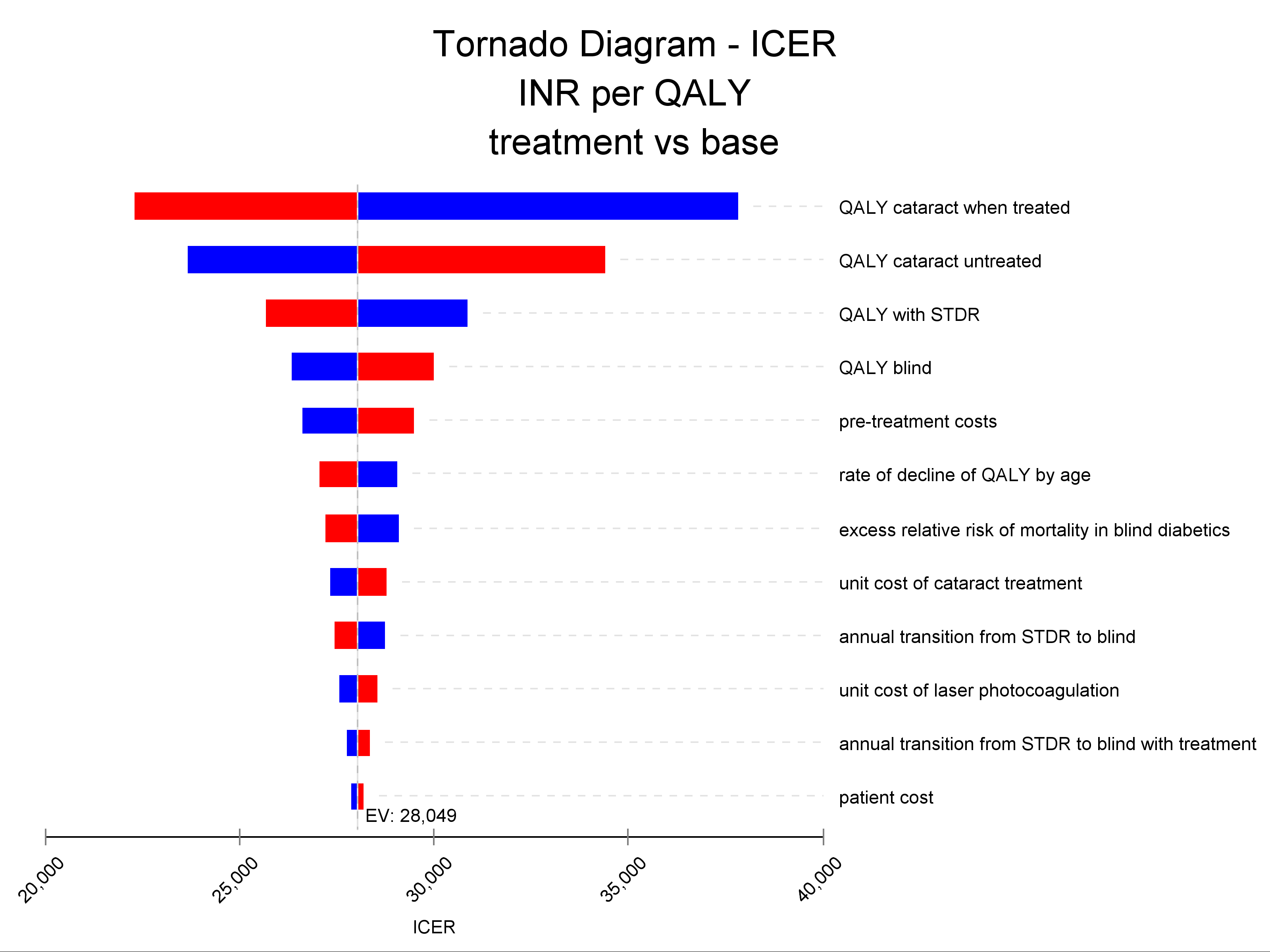


The variables which most affect the incremental cost effectiveness ratio (ICER) are the QALY values of treated and untreated cataract and the QALY values of STDR and blindness (Figure 1). The ICER is less sensitive to the unit costs of screening and of treated and to the relative excess mortality rate among blind people with diabetes.

**Probablistic sensitity analysis (PSA)**

The purpose of the PSA was to test the sensitivity of the ICER to combined uncertainty in all the inputs to estimating its value. Distributions were assigned to each input. The gamma distribution was applied to inputs with a zero lower bound but no upper bound such as unit costs. For the the relative risk of death in blind people with diabetes the gamma distribution was applied to the excess element, that is with an relative risk (RR) of 1.21, the distrbution was applied to 0.21. For inputs whose values lie between zero and unity, such as QALYs and transition rates, a beta distribution was applied.

In each case the variability was represented by a standard deviation (SD) equal to 25% of the point estimate. The large SD value was chosen for two reason: as a demanding test since the central value of the ICER is a fraction of the willingness-to-pay (wtp) threshold and because the basic data does not provide evidence for the distributions.

10,000 Monte Carlo iterations were conducted each of which generates an ICER. A cost-effectiveness sensitivity curve was plotted showing the probability of the ICER exceeding a range of willingness to pay (wtp) thresholds (Figure 2). The probability of meeting a wtp threshold of INR 144,000 (GDP per capita) was just over 82%.

**Figure 2 Cost-effectiveness acceptability curve**

An ICER scattergram was plotted (Figure 3) showing each of the iterations in terms of their QALY gain and cost against the 95% confidence limits and the wtp line. Points the right of and below the wtp line favour the screening programme, while those elsewhere favour the do-nothing option. The choice of a SD of 25% of the mean in the distributions of the individual inputs leads to 18% of the iterations falling on the unfavourable side of the wtp threshold. With an assumed SD of 10%, a lower proportion of iterations would lie on the unfavourable side of the wtp line.

The results based on our chosen assumptions demonstrate the robustness of the result that the programme of screening people with diabetes and treating those diagnosed with STDR or cataract is highly cost effective.

**Figure 3 Incremental Cost-effectiveness: incremental cost v incremental effectiveness**


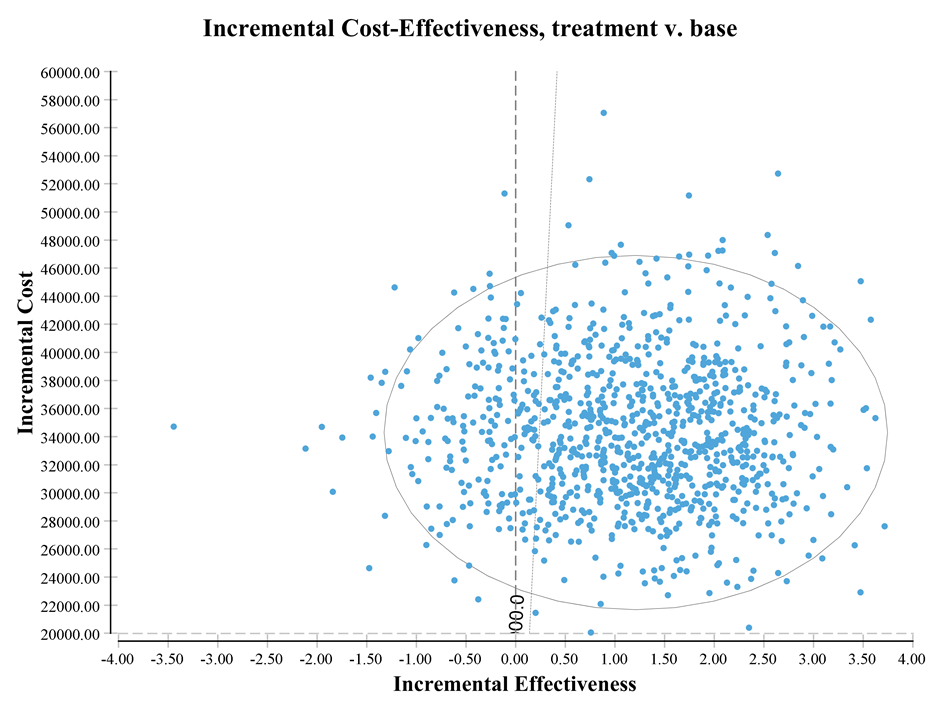


**Figures:**

Figure 1: Incremental Cost-effectiveness Ratio (ICER): sensitivity analysis Tornado diagram

Figure 2: Cost-effectiveness acceptability curve

Figure 3: Incremental Cost-effectiveness: incremental cost v incremental effectiveness
